# Unsupervised Content Mining in CBIR: Harnessing Latent Diffusion for Complex Text-Based Query Interpretation

**DOI:** 10.3390/jimaging10060139

**Published:** 2024-06-06

**Authors:** Venkata Rama Muni Kumar Gopu, Madhavi Dunna

**Affiliations:** Department of EECE, GITAM School of Technology, GITAM Deemed to be University, Rushikonda, Visakhapatnam 530045, India; mdunna@gitam.edu

**Keywords:** CBIR, triplet networks, latent diffusion, content mining, image generation, complex queries

## Abstract

The paper demonstrates a novel methodology for Content-Based Image Retrieval (CBIR), which shifts the focus from conventional domain-specific image queries to more complex text-based query processing. Latent diffusion models are employed to interpret complex textual prompts and address the requirements of effectively interpreting the complex textual query. Latent Diffusion models successfully transform complex textual queries into visually engaging representations, establishing a seamless connection between textual descriptions and visual content. Custom triplet network design is at the heart of our retrieval method. When trained well, a triplet network will represent the generated query image and the different images in the database. The cosine similarity metric is used to assess the similarity between the feature representations in order to find and retrieve the relevant images. Our experiments results show that latent diffusion models can successfully bridge the gap between complex textual prompts for image retrieval without relying on labels or metadata that are attached to database images. This advancement sets the stage for future explorations in image retrieval, leveraging the generative AI capabilities to cater to the ever-evolving demands of big data and complex query interpretations.

## 1. Introduction

The evolution of Content-Based Image Retrieval (CBIR) [1] systems marks a significant transition from conventional methodologies, which used simple queries and image metadata to more advanced prompt mechanisms. Images were used as a query mechanism to input the CBIR systems, as textual queries are blind to certain features of the intended images. With the advent of generative AI, such complex prompt-based queries are no longer limited. Furthermore, input query images may not be available in some applications, where complex descriptions are the only choice. This paper delves into the latest needs of CBIR, focusing on complex text-based queries. Because of their nuanced and multifaceted nature, these queries demand a sophisticated approach to accurately interpret and retrieve relevant images.

In order to tackle this challenge, our research presents a novel framework that leverages the functionalities of Latent Diffusion models [2] to interpret the textual queries. Latent Diffusion, an advanced method in the field of generative AI [3], is the core of the approach, which translates complex textual queries into a content-rich visual representation. Thus, this approach bridges the gap between the query prompt and the user intent. The traditional approach of using keyword-based search miserably fails because of the multi-dimensional nature of the queries, which is often not represented in the keywords.

For the retrieval phase, we utilize the Triplet network architecture [4]. Triplet networks are known for their effectiveness in feature representation and retrieval tasks. We maximize the system’s efficacy by employing triplet networks for the retrieval task.

Our research comprehensively analyzes this approach, exhibiting its effectiveness in handling complex textual queries in CBIR systems. The amalgamation of the Latent Diffusion model with the Triplet network has the prospect of transforming the field of image retrieval. This integration provides a strong and reliable answer to the challenges encountered while understanding intricate queries. This research enhances CBIR technologies and provides opportunities to explore the convergence of unsupervised learning image retrieval in the era of big data and advanced artificial intelligence.

This section highlights the real-world applications of our research area in multiple domains.

Digital libraries and archives [5] utilize complex query processing to enable users to find specific images or documents matching the descriptions they have input. E-commerce platforms [6] can leverage enhanced CBIR systems to improve product search and recommendation engines. In the Healthcare and Medical Imaging industry [7], professionals could make complex queries to find specific types of medical images, for example, scans of particular diseases or conditions to facilitate diagnosis, treatment, and medical education. The most revolutionary application in the surveillance and security domain [8] is the possibility of inputting a complex, descriptive query about a person of interest or a specific activity in a certain location to retrieve the suspect images. Advanced text query-based CBIR systems could help businesses and organizations [9] with extensive digital asset libraries to facilitate the search and retrieval of relevant images. In the Educational platforms and resources [10] domain, complex query CBIR allows educators and learners to employ a more effective way of accessing images that match their learning content as much as possible, be it about historical concepts or scientific topics. Enhanced CBIR systems are helpful in the environmental monitoring framework [11] since scientists can find images with detailed descriptions of the phenomenon. Entertainment and media industry production companies [12] could use the ability to effectively locate particular types of scenes, settings, or character appearances based on extremely complicated descriptions to streamline the production process significantly.

Developing a CBIR system that can precisely handle complex queries requires overcoming multiple challenges. First of all, one of the key obstacles is posed by the semantic gap, as the system has to compare nuanced or multilayered textual descriptions with the images. Even when the information is strictly visual, the data may often include emotions expressed through images, the context of the photograph, or abstract details. As for the nature of the photographic information, it should be noted that the images are highly dimensional, which poses another challenge the system faces. Finally, the effectiveness of the developed system is sensitive to the quality and diversity of data from which it learns. If the inputs to the system are not diverse enough, their processing and results may be biased, rendering such a system unreliable and potentially unfair.

There are several challenges associated with this task when it comes to creating a CBIR system that meets modern applications’ specific demands. For example, many applications require immediate responses, meaning that achieving computational efficiency and real-time processing capabilities presents additional challenges to system design. Building systems capable of responding intuitively is naturally complex, as user queries are often vague and highly subjective. Scalability could be identified as another challenge, as these systems should be capable of functioning with exponentially increasing image databases. Finally, privacy and ethical considerations are of particular concern, as more and more images these days are personal and, in some cases, highly sensitive. The demands of modern applications should be tackled with a multidisciplinary approach.

Our contributions:

1. Problem formulation: We proposed the problem of CBIR, with complex text-based queries as input to the retrieval system.
2. Complex queries: Illustrated some of the complex queries and their multi-dimensional nature that can be leveraged to design a retrieval system.
3. Diffusion models for query interpretation: Explained the diffusion models, focusing on the latent diffusion models and their effectiveness in interpreting complex text queries.
4. Retrieval model: Proposed the custom triplet network model used to retrieve the relevant images from the database.
5. Experimental results: Presented the experimental results, illustrating the effectiveness of the model, and also discussed how we can further enhance the proposed methodology for future works.

## 2. Related Work

This section provides a review of the related work in the field of CBIR systems. The review includes the evolution of the CBIR systems right from the early approaches to the latest deep learning-based approaches, followed by a review of ZS-SBIR systems, and finally a review of the research in the area of diffusion models.

### 2.1. Content-Based Image Retrieval

CBIR is a field of computer vision that deals with the retrieval of pertinent images from a repository of images that rely on the visual content of the images rather than relying on metadata like keywords, tags, or pre-labeled interpretations of the database images. Early approaches in the field utilized features such as color, texture, shapes, and spatial layout [13,14]. Later developments in CBIR used image feature extraction techniques, such as SIFT, binarized statistical image features, and edge descriptors. Various statistical analysis methods and histograms of pixel values are used in [15]. Furthermore, the use of Gabor filters and genetic algorithms has been demonstrated in several studies [16,17,18]. A retrieval approach in the field of remote sensing is presented in [19,20].

More advanced techniques leverage deep learning techniques to achieve the objective. Heba Abdel-Nabi et al. [21] developed an image retrieval method using deep learning by integrating outputs from multiple layers of the AlexNet model to create image representations. Zehra et al. [22] employed an autoencoder to analyze the importance of image blocks for effective retrieval. Similarly, Ashkan et al. [23] implemented a strategy that merges outputs from the fully connected layers of AlexNet with HOG and LBP features, on which PCA was applied for dimensionality reduction to form the final image embedding. The work by [24] presents a two-stage deep learning approach for CBIR, separately optimizing the individual stages through stacked siamese neural networks architecture.

The use of Siamese networks has been proposed to address the orientation issues faced by CBIR systems when using CNNs [25]. Additionally, Yiheng Cai et al. [26] developed a related approach in which the network receives two images as inputs and trains to encode them with features that are similar for akin images and dissimilar for unalike images through a process known as weight sharing. The study by Şaban Öztürk et al. [27] focuses on using a stacked autoencoder in CBIR systems for medical imaging.

### 2.2. Zero-Shot Sketch-Based Image Retrieval (ZS-SBIR)

ZS-SBIR is defined as the process of retrieving relevant images from a database using a sketch as a query without having instances of that particular class included in the training dataset. A graph transformer-based approach is proposed in work [28], which reduces the domain gap through the minimization of Wasserstein distance. A two-part strategy wherein a retrieval module instructs a synthesis module to create various images that progressively adapt to the image domain is proposed by the ACNet framework [29]. Furthermore, [30] developed “StyleGen”, an innovative retrieval strategy for ZS-SBIR that incorporates generating sketch equivalent synthetic images and using stacked siamese neural networks for retrieval.

### 2.3. Diffusion Models

Diffusion models, as referenced in [31,32,33,34,35,36], represent a class of deep generative models that have recently emerged as a major focus within the field of computer vision. These models are known for their remarkable generative abilities, exemplified by the high level of detail and the broad diversity of the examples they generate. Diffusion models have been found to be applicable across an extensive range of generative modeling tasks. These include image generation as cited in [31,32,33,34,35,36], image super-resolution in references [37,38,39,40], and image inpainting noted in [41]. Additionally, they have been used for image editing [42] and image-to-image translation [42], among various other applications. Alternative methods for generating fake samples are studied in [43,44]. Other notable work in image generation achieved through autoregressive models is presented in the work by [45,46].

## 3. Methodology

**Figure 1:**
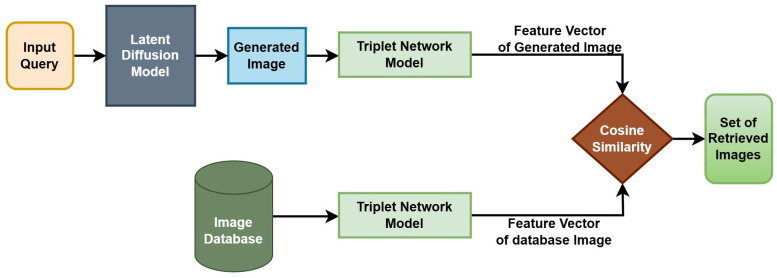
The input query text is fed to the Latent Diffusion model to produce the image equivalent based on the context and details described by the text query, which is further fed to the Triplet Network to obtain the feature representations for the image. The database images are fed through the Triplet network to obtain their equivalent feature representations. A similarity calculator is used to decide the relevant images.

In this section, we explain the complexity of the problem at hand and provide various building blocks of the proposed methodology to tackle the research problem. To the best of our knowledge, this is the first of such works leveraging the Generate AI technique for CBIR tasks. A block diagram illustrating the comprehensive methodology is illustrated in Figure 1.

### 3.1. Problem Formulation

In this section, we set the stage for describing the proposed methodology of CBIR for tackling complex textual queries. We introduce a set of notations to clearly define the key elements involved in the retrieval process, which include the set of queries and the image database.

- Let $Q=\{q_{1},q_{2},\ldots ,q_{n}\}$ denote the set of all complex queries, where each $q_{i}$ represents a textual description encompassing abstract concepts, detailed descriptions, emotions, and contextual information.
- Let $D=\{d_{1},d_{2},\ldots ,d_{m}\}$ represent the set of database images. Here $d_{j}$ denotes *j*th image. This image database is a repository of images from which relevant images are retrieved.
- The objective is to design a function f:Q×D→R, where $R=\{r_{1},r_{2},\ldots ,r_{k}\}$ is the set of images retrieved for a given query $q_{i}$. This function is referred to as the retrieval function and the purpose is to learn and map each of the query $q_{i}$ to a subset of images $r_{k}$ in *D* that are relevant as per the query intent.

### 3.2. Complex Queries

The evolution of the Internet and digital information retrieval landscape has been accompanied by a transformation in the nature of queries, particularly in the context of image retrieval. No longer are queries limited to simple one-word descriptions, such as ‘dog’ or ‘cat’. They have transformed into rather complex text-based descriptions requiring a new interpretation level. These are not class or category labels but descriptions with an image in mind and a clear connotation. For example, ‘A vintage black and white photograph of a group of Beagles participating in a traditional fox hunt, capturing the movement and excitement of the situation’ is one such query that would represent a dog.

What makes the chosen queries so complicated is that they involve various elements, including the topic, the type of image, the action taking place, and even the tone of the image. Thus, the task becomes very complex in the domain of image retrieval. Since the queries include too many different elements, they cannot be processed uniformly and standardly. It needs special consideration as each query is unique and requires a special approach to match the query’s components with the images’ components. The problem is not only that the elements comprising the queries need to be recognized, such as “Beagles” or “fox hunt”. Rather, it is the entire scenario that such text invokes that requires the identification of a range of images where similar scenarios are depicted. Such a high level of information retrieval requires the use of the most advanced technologies and sophisticated query interpretations. It goes far beyond the traditionally used keyword-based search and corresponds to the most sophisticated and innovative AI algorithms.

The following are a few instances of complex text queries that define a dog:

- A high-resolution photograph of a Siberian Husky in a snowy landscape showcasing its thick fur and striking blue eyes.
- An artistic rendering of a Corgi dressed as a medieval knight in a whimsical, storybook illustration style.
- A detailed, close-up portrait of a German Shepherd with a focused expression in a police K-9 vest, set against an urban backdrop.
- A vintage black and white photograph of a group of Beagles participating in a traditional fox hunt captures the movement and excitement.
- A digital painting of a fantasy scene featuring a mythical dog breed with wings and glowing eyes, set in an enchanted forest at twilight.
- A hyperrealistic oil painting of a Labrador Retriever lying on a sunny beach, showing fine details of its wet fur and sand.
- An abstract, cubist interpretation of a Poodle, focusing on geometric shapes and bold colors, reminiscent of Picasso’s style.
- A watercolor scene of a Dachshund in a cozy, home setting, curled up by a fireplace, with soft lighting and warm tones.
- A dynamic, action shot of a Border Collie herding sheep, capturing the motion and energy in a rural, pastoral setting.

Because of the multi-dimensional nature of the queries, the interpretation of the queries is not straightforward. The complexity arises from the subjective nature, abstractness, domain-specific terminology, and contextual descriptions. One way to address this problem is to use advanced Natural Language Processing (NLP) models trained on diverse datasets. However, a complete solution to the text-to-image domain gap can be bridged only through an approach that leverages Natural language understanding together with domain transformation. The proposed approach employs domain transformation through diffusion models, which are presented in the following subsections.

### 3.3. Diffusion Models

Diffusion models are a class of generative AI models that use the diffusion process to gradually add noise to the data and then learn to reverse the process to generate new data samples from noise. The forward process is known as diffusion, and the reverse process is known as generation.

Forward Process: The forward process, also known as diffusion, transforms the initial data distribution $p(x_{0})$ into a Gaussian distribution over *T* timesteps. The process is characterized by a Markov chain that gradually introduces Gaussian noise to the data:

(1) $$p\left(x_{t}|x_{t-1}\right)=N\left(x_{t};\sqrt{1-\beta_{t}}x_{t-1},\beta_{t}I\right)$$

where $\beta_{t}$ is the variance schedule for t=1,…,T, and N denotes the Gaussian distribution.

The forward process can be expressed as follows:

(2) $$x_{t}=\sqrt{\alpha_{t}}x_{0}+\sqrt{1-\alpha_{t}}\epsilon ,\;\epsilon ∼N\left(0,I\right)$$

where $\alpha_{t}=\prod_{s=1}^{t}\left(1-\beta_{s}\right)$ and ϵ is isotropic Gaussian noise.

Reverse Process (Generation): The reverse process learns to reverse the diffusion process starting from noise, gradually denoising it to generate a data sample:

(3) $$p\left(x_{t-1}|x_{t}\right)=N\left(x_{t-1};\mu_{\theta }\left(x_{t},t\right),\Sigma_{\theta }\left(x_{t},t\right)\right)$$

The neural network, parameterized by θ, approximates $\mu_{\theta }\left(x_{t},t\right)$ and $\Sigma_{\theta }\left(x_{t},t\right)$, which represent the mean and variance of the reverse process at each step *t*.

Training Objective: The objective is to minimize the difference between the estimated noise and the actual noise added during the forward process, commonly using the mean squared error:

(4) $$L\left(\theta \right)=E_{x_{0},\epsilon ,t}\left[∥\epsilon -\epsilon_{\theta }\left(\sqrt{\alpha_{t}}x_{0}+\sqrt{1-\alpha_{t}}\epsilon ,t\right){∥}^{2}\right]$$

where $\epsilon_{\theta }$ is the neural network’s estimation of the noise ϵ, given the noised data $x_{t}$ and the timestep *t*.

This training objective teaches the model to reverse the diffusion process by denoising, enabling it to generate new data samples from noise.

### 3.4. Latent Diffusion Models

Latent Diffusion Models (LDMs) extend the foundational theory of diffusion models by applying the diffusion process in a latent space rather than directly in the high-dimensional data space. This approach seeks to address the computational and efficiency challenges of traditional diffusion models.

#### 3.4.1. Autoencoding Phase

The first phase involves compressing data into a latent representation using an autoencoder consisting of an encoder *E* and a decoder *D*. Given an input $x\in R^{D}$, the encoder maps this input to a latent representation $z\in R^{d}$ (with d<D) as follows:

$$z=E(x;\theta_{E}),$$

where $\theta_{E}$ are the parameters of the encoder. The decoder reconstructs the original data from the latent representation:

$$\hat{x}=D\left(z;\theta_{D}\right),$$

with $\theta_{D}$ denotes the parameters of the decoder. The autoencoder is trained to minimize the reconstruction error, often using the mean squared error (MSE) loss:

$$L_{AE}\left(\theta_{E},\theta_{D}\right)={∥x-\hat{x}∥}_{2}^{2}.$$

#### 3.4.2. Diffusion Process in Latent Space

After encoding the data, the diffusion process is applied to the latent representations. The forward process incrementally adds Gaussian noise to z, transforming it into a noise distribution:

$$z_{t}=\sqrt{\alpha_{t}}z+\sqrt{1-\alpha_{t}}\epsilon ,\;\epsilon ∼N\left(0,I\right),$$

where $\alpha_{t}$ decreases to 0 as *t* approaches *T*.

The reverse process seeks to reconstruct the latent representation from the noise:

$$z_{t-1}=\frac{1}{\sqrt{\alpha_{t}}}\left(z_{t}-\frac{1-\alpha_{t}}{\sqrt{1-\alpha_{t}}}\epsilon_{\theta }\left(z_{t},t\right)\right),$$

where $\epsilon_{\theta }\left(z_{t},t\right)$ is the model’s prediction of the noise added at timestep *t*. The model is trained to minimize:

$$L_{LDM}\left(\theta \right)=E_{z,\epsilon ,t}\left[∥\epsilon -\epsilon_{\theta }\left(z_{t},t\right){∥}_{2}^{2}\right].$$

#### 3.4.3. Generating New Samples

To generate new samples, start with $z_{T}∼N\left(0,I\right)$ and apply the reverse process to denoise it to $z_{0}$, then reconstruct the data sample:

$$\hat{x}=D\left(z_{0};\theta_{D}\right).$$

LDMs leverage the latent space’s dimensionality reduction alongside diffusion models’ generative capability for efficient, high-quality generation across various applications.

### 3.5. Transforming Complex Queries into Images Using LDMs

The three main components in LDMs are an Autoencoder(VAE), a U-Net, and a text-encoder.

- Autoencoder(VAE): The VAE architecture [47] consists of two main components: an encoder and a decoder. The encoder compresses the image into a condensed latent space representation, which is then fed into the U-Net model. On the other hand, the decoder works to expand this latent representation back into its original image form. In the training phase of latent diffusion, the encoder extracts latent representations (or latents) from images to initiate the forward diffusion process, progressively adding noise with each step. Conversely, during inference, the cleaned latents produced by the reverse diffusion are reconstructed into images by the VAE decoder. It is important to note that the VAE decoder is the only component required during the inference process.
- U-Net: The U-Net architecture [48] is structured into two sections: an encoder and a decoder, both of which utilize ResNet blocks. The encoder’s function is to downscale an image into a reduced resolution format, while the decoder reverses this process, aiming to restore the image to its original, higher resolution form, ideally with reduced noise. Specifically, the output of the U-Net is designed to forecast the noise residual, facilitating the generation of a refined, denoised image representation. Shortcut connections are integrated to ensure critical information is not lost during the downsampling process, linking the downsampling ResNets in the encoder directly to the upsampling ResNets in the decoder. Moreover, the latent diffusion version of the U-Net incorporates the ability to tailor its output based on text embeddings, achieved through the incorporation of cross-attention layers. These layers are strategically placed within both the encoder and decoder segments, typically amidst the ResNet blocks.
- Text-encoder: The text encoder is responsible for converting the input prompt into an embedding space that can be comprehended by the U-Net. Typically, a basic transformer-based encoder is used to convert a series of input tokens into a series of latent text embeddings. A pre-trained CLIP model [49], known as CLIPTextModel, is used as a text encoder.

**Figure 2:**
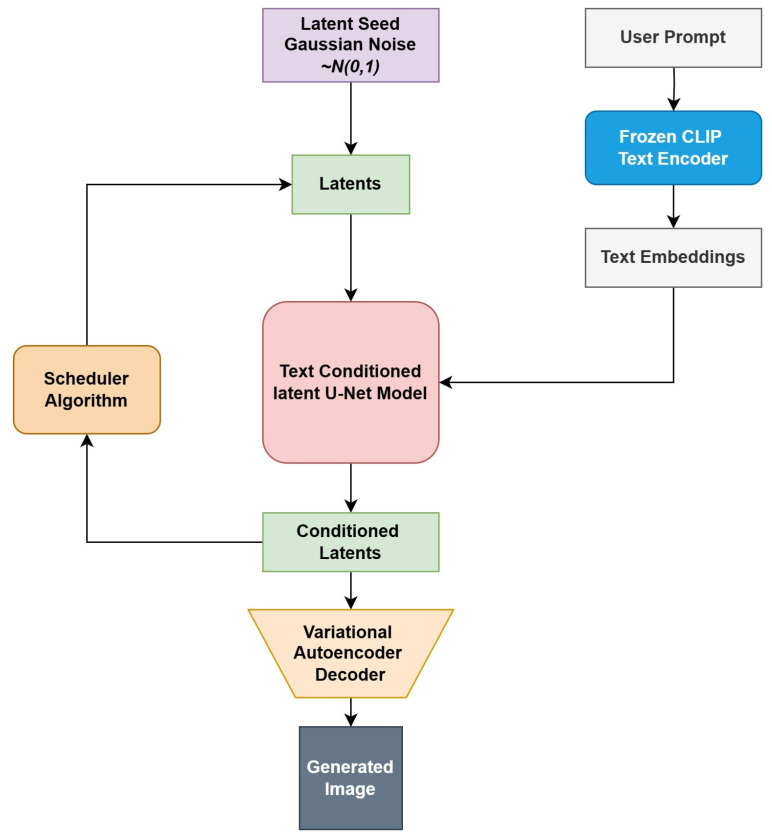
The latent diffusion model operates by accepting two inputs: a latent seed and a text prompt. The latent seed initiates the creation of randomized latent image representations with dimensions of 64 × 64, while the text prompt is converted into text embeddings measuring 77 × 768 through the use of CLIP’s text encoder. Following this, the U-Net methodically removes noise from the random latent image representations, taking guidance from the text embeddings. The U-Net’s output, which represents the noise residual, is then employed alongside a scheduling algorithm to derive a cleaned-up version of the latent image representation.

The block diagram of the logical flow in inference is presented in Figure 2.

**Figure 3:**
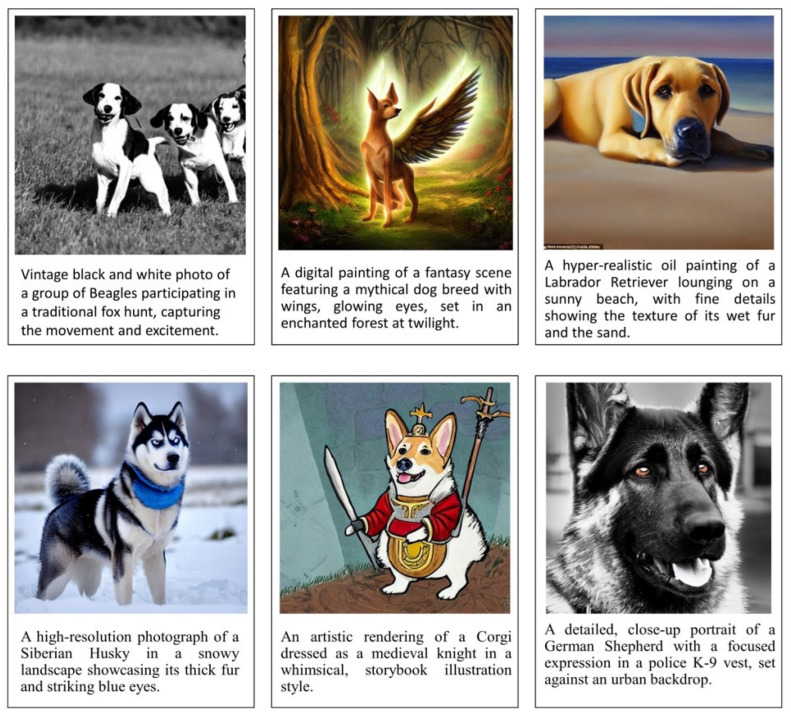
Samples of images generated when the complex queries are given as input to the diffusion model.

The images generated using the Latent Diffusion Model are presented in Figure 3.

### 3.6. Domain-Gap Problem

In addressing the challenge of Content-Based Image Retrieval (CBIR) with complex textual queries, the approach innovatively leverages the capabilities of Diffusion models to generate query images from textual descriptions. While this approach is robust enough to cater to a wide range of complex queries, which bridges the semantic gap between the user intent and the images in the database, it can potentially cause domain gap problems. The reason is that the generated images differ from the database images in various aspects such as texture, color distribution, quality, etc. To combat this, domain adaptation techniques can be employed through re-training the diffusion models to align the feature spaces of the generated and the database images. However, in this work, we are not scoping these and this can be explored as future work that extends this current research.

### 3.7. Image Retrieval Using Triplet Networks

We employ triplet networks for the image retrieval portion of the proposed CBIR system. Triplet network architecture is a specialized deep-learning architecture where three images, known as anchor, positive, and negative images, are used to train the network to learn the similarities and differences between various samples in the dataset. In this, the anchor is a reference image, a positive is a sample similar to the anchor, and a negative is one that is dissimilar to the anchor. A loss function known as triplet loss is used to train the network.

- Anchor: An anchor is a reference sample that will be used to compare and reference other data points in the database.
- Positive: A positive is a data sample that is similar to the anchor.
- Negative: A negative is a data point or image dissimilar to the anchor.

During the training process, the triplet network would be presented with the sets of these three types of data points, i.e., (*anchor*, *positive*, *negative*). The objective of the training process is to encode the images in such a manner that the anchor and the positive are closer in the feature space compared to the anchor and the negative. In order words, the anchor and positive samples should be pulled together in the feature space, while the anchor and negative samples are pushed apart through the learning process.

#### 3.7.1. Triplet Loss Function

The loss function utilized for training triplet networks is known as the *Triplet Loss*. The function aims to ensure that the distance between anchor and positive samples is always greater than between anchor and negative samples. This triplet loss is very effective in comparison with the contrastive loss function, which Siamese neural networks use because of its ability to handle difficult samples in the datasets.

In mathematical terms, the Triplet Loss can be formulated as:

(5) $$L_{\mathrm{Triplet}}=\max\left(0,d\left(a,p\right)-d\left(a,n\right)+\mathrm{margin}\right),$$

where:

- $L_{\mathrm{Triplet}}$ denotes the loss associated with the triplet.
- d(a,p) denotes the Euclidean distance between the anchor (*a*) and the positive (*p*) in the feature space.
- d(a,n) is the distance of the negative (*n*) from the anchor (*a*).
- margin delineates the minimum separation between the positive and negative pairs, adding to the robustness of the embedding. This is a hyperparameter.

The usage of the max function ensures the loss remains non-negative and that the model incurs no penalty if the negative example is adequately distanced from the positive relative to the anchor beyond the predefined margin. Effectively, the loss transitions to zero if d(a,p)+margin<d(a,n), indicating the model’s accurate positioning of the positive closer to the anchor than the negative, exceeding the margin. The objective during the training process is to minimize this loss across all feasible triplets in the dataset.

#### 3.7.2. Training of Triplet Network

The process of assembling training data for triplet networks involves creating triplets of image, categorized as (a, p, n). In this context, ‘a’ denotes the anchor, ‘p’ stands for the positive, and ‘n’ represents the negative. This selection strategy is crucial for effectively training the network to distinguish between similar (positive) and dissimilar (negative) images relative to an anchor. The steps for triplet formation are outlined as follows:

- Begin with the random selection of two distinct classes from the total available classes. Assign one as the positive class and the other as the negative class.
- From the collection of images, randomly select two of them from the positive class. These are designated as the anchor and positive instances for the triplet.
- Subsequently, choose a single image from the negative class at random. This serves as the negative instance, completing the triplet for the training dataset.

This method ensures a diverse range of triplets, challenging the network to learn robust distinctions between images based on class similarity and dissimilarity. This process will be carried out for a series of iterations that match the batch size, and the generated triplets will be input into the neural network to perform the training phase. After the triplets are formed, they are input into the sub-network. The output generated by these sub-networks for each triplet is utilized to calculate the triplet loss. This loss is subsequently employed to update the model’s weights via the gradient descent algorithm. For the implementation, we employ a modified version of the triplet loss, which will be detailed below. This variant slightly deviates from the one described in earlier sections.

Let Φ denote the total transformation function performed by the neural network. Let $A_{out}$, $P_{out}$, and $N_{out}$ represent the outputs of the network corresponding to the Anchor, Positive, and Negative inputs, respectively, when transmitted through the network as part of a triplet.

(6) $$\begin{array}{cc} A_{out} & =\Phi (\mathrm{Anchor}\;\mathrm{Sample}) \end{array}$$

(7) $$\begin{array}{cc} P_{out} & =\Phi (\mathrm{Positive}\;\mathrm{Sample}) \end{array}$$

(8) $$\begin{array}{cc} N_{out} & =\Phi (\mathrm{Negative}\;\mathrm{Sample}) \end{array}$$

We initially compute the Manhattan distances between the anchor output and both the positive and negative outputs.

(9) $$\begin{array}{cc} \; & PositiveDistance\left(PD\right)=|A_{\mathrm{out}}-P_{\mathrm{out}}{|}_{1} \end{array}$$

(10) $$\begin{array}{cc} \; & NegativeDistance\left(ND\right)=|A_{\mathrm{out}}-N_{\mathrm{out}}{|}_{1} \end{array}$$

Here, ${\left|\cdot \right|}_{1}$ refers to the L1 distance, also known as the Manhattan distance. The calculated distances are then converted into probabilities using the softmax function.

(11) $$p_{+}=\frac{e^{PD}}{e^{PD}+e^{ND}}$$

(12) $$p_{-}=\frac{e^{ND}}{e^{PD}+e^{ND}}$$

Here, $p_{+}$ and $p_{-}$ represent the probability equivalents of the positive and negative distances, respectively. The definition of triplet loss is given below:

(13) $$L_{\mathrm{Triplet}}=Mean(|p_{+}|+|1-p_{-}\left|\right)$$

The following are the hyper-parameters of the training process:

- Optimizer: Adam optimizer
- Learning rate: 0.001
- Epsilon: 0.0000001
- Number of epochs: 30
- Steps in epoch: 1000
- Validation steps: 200
- Batch size: 64
- Regularization: Drop out
- Dropout rate: 0.25

#### 3.7.3. Prediction and Image Retrieval

Once training has concluded, the final layer of the sub-network serves as the feature representation for the input images. The retrieval process involves generating feature representations for the diffusion model-generated query image and each image within the database. Cosine similarity is employed as the metric of choice to identify and rank images based on their relevance to the query.

**Definition** **1.** *Cosine similarity between two vectors α and β can be defined using the following formula:*

(14) $$\mathrm{Cosine}\;\mathrm{Similarity}=\frac{\alpha \cdot \beta }{∥\alpha ∥∥\beta ∥}$$

*α·β represents the dot product of vectors α and β.*

*∥α∥ is the Euclidean norm of vector α.*

*∥β∥ is the Euclidean norm of vector β.*

#### 3.7.4. Steps in Image Retrieval

The overall image retrieval process can be summarized as below:

1. Pass the complex text query to the diffusion model to create an equivalent image representation.
2. Resize the image to match the dimensions of the database images.
3. Pass the database images through the trained triplet network model to create the corresponding feature representations.
4. Pass the query image representation through the triplet network model to create its feature representation.
5. Evaluate the cosine similarity between the query feature representation and those of the database.
6. Rank the images based on the similarity to publish the retrieved top relevant images.

### 3.8. Network Architecture of Triplet Network

**Figure 4:**
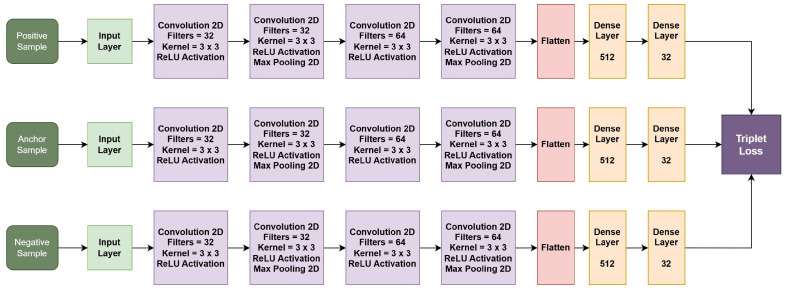
Network architecture of Triplet network.

The network architecture for each branch of our triplet network is detailed in Table 1. This structure is critical for processing the input data through the network’s parallel branches, which are designed to handle anchor, positive, and negative samples uniformly. Table 1 lists each layer’s specifications, including its type, configuration, and the dimensions of its output, offering a clear view of our network’s setup. Table 2 and Figure 4 present the network architecture and configuration of the overall triplet network, combining anchor, positive, and negative branches.

The following Table 3 is the evaluation of image retrieval on the test set of the CIFAR-10 dataset:

## 4. Experimental Results

This section extensively discusses the experimental results based on the methodology we have chosen to implement within the Complex text query image retrieval domain. The dataset against which we conduct our evaluation is the CIFAR-10 dataset, which is used as the retrieval database. The Latent Diffusion model used for the experiments is a pre-trained text-to-image latent diffusion model developed by Rombach et al. [2], trained on samples from the LAION-5B dataset [50]. The model uses a frozen CLIP ViT-L/14 text encoder to incorporate textual instructions. It is a lightweight model, as the components are 860M U-Net and 123M text encoder.

### 4.1. Datasets

The dataset used as the retrieval database is the CIFAR-10 dataset. The dataset is composed of 60,000 images distributed over 10 different categories. The dimension of the images in the dataset is 32×32; 10,000 images from the dataset are sampled with equal representation for the ten classes for the experimentation. The data are divided into the ratio of 85:15, where 85% of samples are used for training the triplet network model, while the rest of 15% images were used for validation and fine-tuning the model.

### 4.2. Evaluation Metric

We use Mean Average Precision (mAP) as a measure of how well the CBIR system handles complex text-based queries. The mean Average Precision (mAP), a numerical value, gives the average precision across various queries. First, each text query’s Average Precision (AP) is computed, and these APs are averaged. The mean Average Precision (mAP) is a reliable and popular way to measure how well retrieval systems work overall.

Specifically, mAP@25 is used in the experiments, which is read as mean average precision at rank 25. It measures the mean of the average precision scores across all queries, considering only the top 25 retrieved items. For the evaluation of mAP@25, the following steps are followed: For each query, retrieve the top 25 items and, based on the relevancy, compute the precision for each individual query. Next, compute the average precision for each query by averaging the precision values obtained at each relevant item position. Finally, average the average precision scores across all queries to obtain mAP@25. While ranking the top relevant images, the numerical values of the similarity scores are used for sorting and ranking.

Mean Average Precision at 25 (MAP@25) is given by:

(15) $$\mathrm{mAP}@25=\frac{1}{\left|Q\right|}\sum_{q\in Q}\mathrm{AP}@25\left(q\right)$$

where AP@25(q) for a single query *q* is defined as follows:

(16) $$\mathrm{AP}@25\left(q\right)=\frac{1}{\left|R\right|}\sum_{k=1}^{25}P\left(k\right)\times \mathrm{rel}\left(k\right)$$

|Q|: Number of queries.

|R|: Number of relevant items for the query.

P(k): Precision at rank *k*.

rel(k): Its value is 1 if the item at rank *k* is relevant, and 0 otherwise.

### 4.3. Performance Comparison

**Figure 5:**
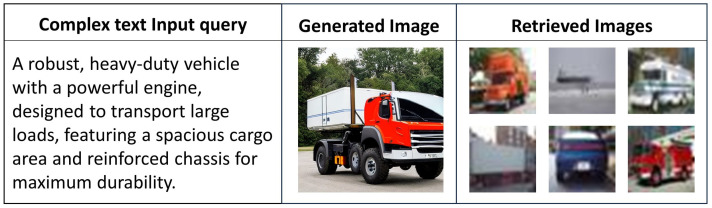
Sample output of a retrieval task.

In this study, we compared the performance of our proposed Advanced Complex Text Query-based CBIR system, which incorporates a latent diffusion model and triplet networks, to typical text-based image retrieval systems. The results Table 4 clearly show that our suggested system outperforms standard text-based picture retrieval methods. This achievement was made possible by the unique ability of the latent diffusion model to generate high-quality image representation from an accompanying text description, as well as the triplet network, which efficiently and effectively learns the derived fine-grained image differences. It was also observed that our system is resistant to various query complexities. In this case, even under the worst-case scenarios of having extremely dense and abstract queries, our system still performed more or less in the same way as it did when the queries were less complex. Currently, the main limitation of the existing text-based image retrieval methodologies is the reliance on the exact matching of keywords or key expressions. As such, these technologies tend to be extremely rudimentary, to the point that they cannot afford to process the queries through semantic perspectives. As a result, traditional methods often provide users with a large percentage of images that vary in contextual relevance. Our system tackles this issue head-on due to its usage of advanced modeling tools that were leveraged to identify, interpret, and represent the hidden meanings contained in the text. A sample visual retrieval result is presented in Figure 5. In our comparison, we use Big-O notation to express the time complexity of each approach, where O(r) denotes the retrieval time and O(r+g) denotes the combined retrieval and generation times, highlighting the additional computational overhead in the proposed approach.

The approaches used in performance comparison are as follows: Keyword-based retrieval uses certain keywords or annotations to retrieve images [51]. The other approach uses a specialized GAN known as Text Conditioned Auxiliary Classifier Generative Adversarial Network (TAC-GAN) [52] for text-to-image generation. A Natural Language Processing (NLP) [53] based approach leverages NLP for transforming complex text queries into features through which the label is inferred for image retrieval.

### 4.4. Ablation Studies

#### 4.4.1. Retrieval Method Selection

In this subsection, we present the experimental comparison of two different retrieval approaches: triplet networks and autoencoders. These experiments aimed to determine the most effective method for our image retrieval task. We conducted extensive experiments using both methods on our dataset, and the evaluation metric used is Mean Average Precision at 25 (mAP@25). The experiments in Table 5 indicate that triplet networks are more effective for image retrieval. The ability of triplet networks to directly optimize the embedding space for similarity tasks gives them a significant advantage over autoencoders, making them the preferred choice for our retrieval system.

#### 4.4.2. Loss Function Selection

Experiments were conducted to select the appropriate loss function among contrastive and triplet loss functions. The results of the experiments in Table 6 indicate that the triplet loss function is more effective for image retrieval in the current framework of complex text query-based image retrieval. The reason for the better performance of triplet loss is its ability to handle difficult samples in the data.

##### Limitations and Areas for Improvement

- The current implementation utilizes a pre-trained model to generate images from complex text queries without the need for retraining or adapting it to the database’s domain. Although this method is feasible, its effectiveness may be enhanced because we sometimes have knowledge about the database’s domain. Hence, a possible future course of action to enhance the established system would be to optimize it for the particular database it is utilized with.
- Another important constraint of our method pertains to ethical problems linked to the utilization of Generative AI models. Although these algorithms can generate highly realistic images based on text descriptions, they can unintentionally continue or magnify biases in the data used for training. This can result in the creation of stereotyped or culturally inappropriate images, distorting the intended message and perpetuating damaging stereotypes. Moreover, the capacity of these models to produce authentic images gives rise to apprehensions over the possibility of their misuse, such as the creation of misleading or deceptive visuals.

## 5. Conclusions

In conclusion, our study presents a novel, Advanced, Complex Text Query-based CBIR system that leverages a latent diffusion model combined with triplet networks to significantly enhance the accuracy and efficiency of image retrieval from complex text queries. The proposed system outperforms traditional text-based image retrieval methods, demonstrating a higher mean average precision and improved retrieval efficiency. This advancement underscores the potential of integrating sophisticated machine learning models to handle the nuances of complex queries effectively.

## Figures and Tables

**Table 1:** Network architecture and configuration of sub-networks of triplet network.

Layer	Configuration	Output Shape	Parameters
Convolution 2D	Filters: 32 Kernel = 3×3 padding = same	32×32×32	896
Activation	ReLU	32×32×32	0
Convolution 2D	Filters: 32 Kernel = 3×3	30×30×32	9248
Activation	ReLU	30×30×32	0
Max Pooling 2D	pool_size: 2×2	15×15×32	0
Dropout	0.25	15×15×32	0
Convolution 2D	Filters: 64 Kernel = 3×3 padding = same	15×15×64	18,496
Activation	ReLU	15×15×64	0
Convolution 2D	Filters: 64 Kernel = 3×3 padding = same	13×13×64	36,928
Activation	ReLU	13×13×64	0
Max Pooling 2D	pool_size: 2×2	6×6×64	0
Dropout	0.25	6×6×64	0
Flatten		2304	0
Dense		512	1,180,160
Activation	ReLU	512	0
Dense		32	16,416

**Table 2:** Network architecture and configuration of triplet network.

Layer	Output Shape	Parameters
Input Layer 1	32×32×3	0
Input Layer 2	32×32×3	0
Input Layer 3	32×32×3	0
Sequential	32	1,262,144
Vectors (Concatenate)	96	0

**Table 3:** Performance evaluation of custom triplet network.

Class	Precision	Recall	F1-Score	Support
Aeroplane	0.56	0.58	0.57	1000
Automobile	0.59	0.59	0.59	1000
Bird	0.49	0.47	0.48	1000
Cat	0.52	0.51	0.51	1000
Deer	0.38	0.37	0.37	1000
dog	0.46	0.47	0.46	1000
Frog	0.49	0.48	0.48	1000
Horse	0.6	0.58	0.59	1000
Ship	0.59	0.66	0.62	1000
Truck	0.57	0.61	0.59	1000

**Table 4:** Performance comparison of proposed methodology in comparison with existing approaches.

Approach	mAP@25	Retrieval Time Complexity
Keyword-based retrieval	5.7	O(r)
TAC-GAN	19.6	O(r)
NLP model approach	29.3	O(r)
Proposed methodology	45.2	O(g+r)

**Table 5:** Retrieval method assessment.

Approach	mAP@25
Autoencoder	22.4
Triplet Network	45.2

**Table 6:** Loss function assessment.

Approach	mAP@25
Contrastive Loss	39.5
Triplet Loss	45.2

## Data Availability

The original data presented in the study are openly available at https://www.cs.toronto.edu/~kriz/cifar.html (accessed on 1 February 2024).
